# Meeting learners where they are: case presentations in remote pediatric critical care education

**DOI:** 10.3389/fped.2026.1895772

**Published:** 2026-08-04

**Authors:** Adeolu Aromolaran, Ruchit Nagar, Claudia Adja-Sai, Renee Alce, Tigist Bacha, Shitahun Fentie, Marie Charlyne Kilba, Sheendy Lilite, Brian S. Marcus, Marguereth Mehu, Aneso Mohammed, Uchenna Nnajekwu, Odiraa Nwankwor, Chiesonu Nzeduba, Larko Owusu, John Adabie Appiah, Frank Owusu-Sekeyre, Tagbo Oguonu, Priya Prabarkaran, Andrew Wu, Adam M. Silverman, Ayanaw Tamene, Charles Udemba, Bitseat W. Haile, Susan Zakariah, Michael F. Canarie

**Affiliations:** 1 Department of Pediatrics, Rutgers Robert Wood Johnson Medical School, New Brunswick, NJ, United States; 2 Department of Pediatrics, Yale School of Medicine, New Haven, CT, United States; 3 Department of Pediatrics, The University of British Columbia, Vancouver, BC, Canada; 4 Department of Pediatrics, Hôpital Universitaire La Paix, Port-au-prince, Haiti; 5 Department of Pediatrics, St. Paul Hospital Millennium Medical College, Addis Ababa, Ethiopia; 6 Department of Pediatrics, Bahir Dar University College of Science, Bahir Dar, Ethiopia; 7 Department of Pediatrics, Greater Accra Regional Hospital, Accra, Ghana; 8 Department of Pediatrics, Hopital Saint-Damien, Port-au-Prince, Haiti; 9 Department of Pediatrics, Albert Einstein College of Medicine, New York, NY, United States; 10 Department of Pediatrics, Jimma University, Jimma, Ethiopia; 11 Department of Pediatrics, University of Nigeria Teaching Hospital, Ituku, Nigeria; 12 Department of Pediatrics, Nemours Children’s Hospital, Wilmington, DE, United States; 13 Department of Pediatrics, Cooper University Hospital, Camden, NJ, United States; 14 Department of Pediatrics, Enugu State University Teaching Hospital, Parklane, Nigeria; 15 Department of Pediatrics, Komfo Anokye Teaching Hospital, Kumasi, Ghana; 16 Department of Pediatrics, Korle Bu Teaching Hospital, Accra, Ghana; 17 Department of Pediatrics, The University of Alabama at Birmingham, Birmingham, AL, United States; 18 Department of Pediatrics, Hennepin Healthcare, Minneapolis, MN, United States; 19 Department of Pediatrics, University of Connecticut School of Medicine, Farmington, CT, United States; 20 Department of Pediatrics, Black Lion Hospital, Addis Ababa, Ethiopia

**Keywords:** case based learning, child mortality, global child health, low- and middle-income countries, medical education—clinical skills training, pediatric critical care education, resource- limited settings

## Abstract

As the burden of pediatric critical illness is heaviest in low- and middle-income countries (LMIC), educating trainees in recognizing and treating the sickest children may attenuate high mortality rates, morbidity and hospital length of stay. Remote education is increasingly utilized to provide effective teaching across borders. We describe the efforts of an international collaborative group created to supplement pediatric critical care education through online didactics. An essential part of this has been integrating case presentations into the curriculum to supplement traditional topic-based teaching. Ninety-five case presentations have been performed, of which acute respiratory failure, infectious disease without sepsis and neurologic disease were the most common pathologies discussed. In addition, we considered issues such as the applicability of guidelines developed in high-income countries (HIC) and the ethical complexity of caring for critically ill children in LMIC, which often have limited resources. Case presentations also allow a deeper appreciation of the circumstances in which colleagues provide care for critically ill children in RLS and foster bidirectional learning. Discussion of cases with international faculty can enrich discussions, strengthen global bonds and promote collaboration among the larger community.

## Introduction

The inequity in global healthcare for children has been well documented, and childhood mortality rates are markedly higher in low- and middle-income countries (LMIC) than in more affluent regions of the world (1, 2). The reasons for this imbalance are multifactorial, but insufficient numbers of trained physicians contribute to the problem (3). Since nearly half of hospitalized pediatric deaths in LMICs result from acute and critical illness, the lack of expertise and resources to educate trainees in the recognition and care of severely ill children is highly relevant (4). In-country training programs are developing across sub-Saharan Africa, and international partnerships can effectively bolster the educational framework to address this critical gap (5). Advances in teleconferencing software and broadband technology have made remote education practical, cost-effective and widely accessible in many low-resource settings (6). Although evidence is limited, e-learning modalities may offer similar knowledge retention and learner satisfaction when compared to traditional methods (7). We describe a remote, case-based learning program developed with partners in LMICs, and discuss some of the lessons learned from this initiative.

## Global partnerships: pediatric critical care in resource-limited settings (PCCiRLS)

Pediatric Critical Care in Resource-Limited Settings (PCCiRLS) is a collaborative remote pediatric critical care (PCC) teaching program established by volunteer clinician educators with experience working and teaching in LMICs. The program began in 2021 in partnership with St. Damien Pediatric Hospital in Port-au-Prince, Haiti, and has grown to include 13 facilities in Haiti and Sub-Saharan Africa. Contributors and moderators come from 19 countries (high-income countries and LMICs), across four continents. Early in the program, case presentations were incorporated into the curriculum at the request of partners and have since become an integral part of the curriculum. PCCiRLS has conducted nearly 450 biweekly teaching sessions since 2021, of which 95 have featured case presentations.

## Case presentations

Case-based learning (CBL) is a well-established pedagogical method that helps bridge theoretical frameworks with real-world clinical scenarios. It is well-suited to adults for whom learning is integrated into experiences and reinforced by its relevance (8, 9). CBL also promotes more active participation in learning, leading to more trainee engagement, critical thinking, and knowledge retention consistent with adult cyclical learning (8).

We incorporated case presentations to leverage our international faculty to take advantage of the benefits of CBL. Any medical teaching program involving exchanges across regional and cultural spans should surely question whether the discussions are relevant to all parties. In this context, case presentations have permitted the global faculty insight into these on-the-ground realities–to meet the learners where they are. These presentations bridge gaps in understanding and experience. As a result, *practical* discussions on patient care focus on how material gaps are accommodated and on therapies that are both feasible and effective in the setting. It is an educational encounter in which *all participants* can learn and share their experiences.

Cases are prepared by trainees at partner sites under faculty guidance. Currently, nine teaching hospitals across Nigeria, Ghana, Haiti, and Ethiopia prepare and host case presentations. The frequency of presentations is determined by the host sites (every 3–6 sessions or 6–12 weeks), interspersedwith more formal didactic sessions. Case presentations often focus on complex or challenging cases that invite a review of fundamental principles in pediatric critical care. Presenters review the caseand hospital course, followed by a discussion of the salient clinical or ethical questions with a review of relevant literature.

## The cases

**Figure 1:**
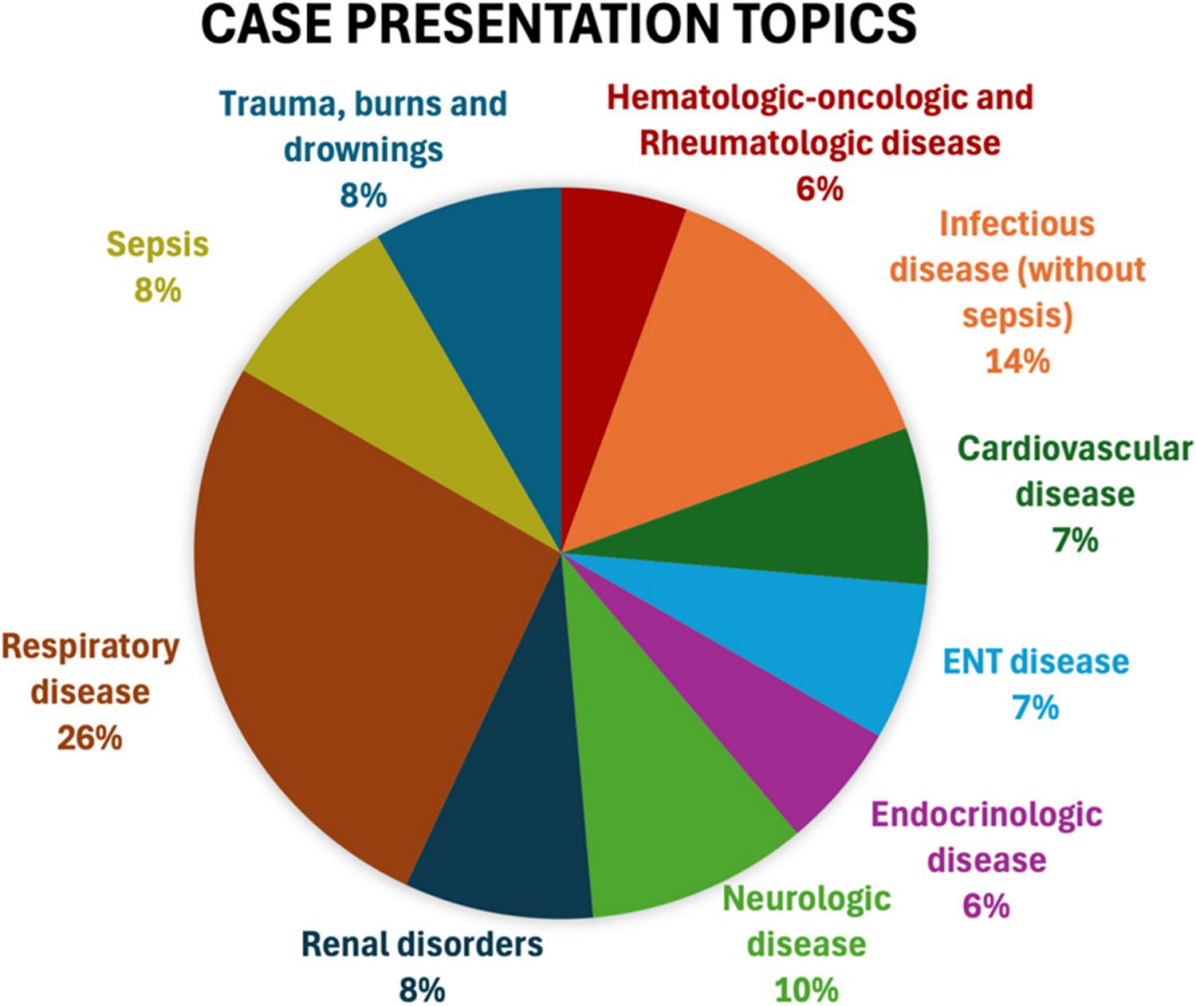
Distribution of primary illness in PCCiRLS case presentations. Shown are the categories of primary illness for case presentations from 2021 to 2025 as defined by the presenting clinical team.

The cases comprise a broad range of critical illnesses (Figure 1). We characterized cases by the primary diagnosis at admission. However, overlapping pathologies and complications were common, and many patients presented or developed multiorgan dysfunction. Many of the patients also have multiple comorbidities, such as severe acute malnutrition (SAM) and anemia. Respiratory pathology is the most common presenting illness [with pediatric acute respiratory distress syndrome (PARDS) as the largest segment], followed by infectious disease without sepsis and neurological dysfunction (principally infectious and traumatic). This spectrum is consistent with prior studies evaluating the causes of pediatric acute illness in LMIC, with the notable exception of sepsis (5). While sepsis is a leading cause of acute illness and mortality worldwide, there were infrequent case discussions regarding children presenting with fulminant sepsis. We believe this is likely due to a bias towards selecting uncommon or extreme cases for discussion. It is clear from many of the cases that timely access to appropriate care plays a significant role in outcomes. This is particularly true in cases of airway obstruction, where prompt presentation and swift interventions lead to positive outcomes. Sadly, delays in presentation contribute to poor results, particularly in children with CNS infections. Several broad, recurring themes have emerged throughout the program.

## The case setting: social, financial, and ethical challenges in pediatric critical care

Global income inequality and the concomitant social, environmental, and political factors that undermine public health are the backdrop to the cases discussed in this forum. These disparities undermine child health in a variety of ways: limited access to healthcare; limited health awareness; inadequate infrastructure, sanitation and housing; and other socio-economic challenges. Superimposed on these are the concrete health system resource constraints and insufficient acute and critical care capacity. Regarding the care of the sick child, fragile referral and patient transport systems contribute to sicker patients on presentation. There are also a finite number of ICU beds available to accommodate the critically ill child; beds that may only be available to those most severely–– perhaps irretrievably–ill. Lastly, delays due to resource limitations extend to families’ inability to finance crucial materials for clinical testing and treatment in a timely fashion.

Inequities in civil infrastructure can lead to serious health consequences, as exemplified by surges in motor-vehicle injuries and incidents related to other accidents and natural disasters (10). For instance, one case involved a child who fell into an open sewer, suffering a submersion injury in contaminated water—a situation further complicated by insufficient resuscitative capacity at the presenting hospital. Unlike high-income countries, where drownings are almost exclusively associated with recreation, such incidents in LMICs often reflect deeper social determinants, including inadequate sanitation and public works or lack of regulatory oversight (11).

Specific clinical cases vividly illustrate these challenges. Over four months, three different sites reported cases of laryngeal papillomatosis requiring emergent surgical excision—a condition that could have been prevented by maternal vaccination (12). Moreover, the inability to perform preferred surgical approaches and/or adjunctive medical management may increase complications and recurrence rates. We noted numerous cases of unintended extubations as well; these are often associated with patient agitation, sedation, night shifts, nursing ratios and experience (i.e., resources and training) (13). The group discusses ethical dilemmas. Clinicians often grapple with the allocation of scarce resources amid high demand. A particularly complex ethical situation involved a toddler with meningoencephalitis who presented in refractory status epilepticus that progressed into cessation of all neurological activity. This case raised critical questions regarding the accurate diagnosis of brain death when ancillary equipment or training is lacking, as well as the implications of such a diagnosis in the absence of legal frameworks that codify brain death. These regulatory gaps complicate the ethical landscape for clinicians, deny PICU services for those who might benefit the most, and impact families’ understanding of brain death and end-of-life decision-making (14).

## Variations in practices in LMIC

There is a wide variation in practice revealed by these cases, a function of differing regional approaches, experiences, and resource constraints. Case presentations allow us to discuss management strategies, examining variation in practices between LMIC and HIC, and questioning which of the practice patterns were truly evidence-based, feasible, and appropriate. What is clear is that the populations and diseases treated differ greatly between the two settings. It is important to acknowledge that, as compared with adults, pediatric intensive care has fewer evidence-based clinical guidelines–even in HICs–further compounding clear case management in LIC and LMICs. Such situations complicate the adaptation of guidelines to fit local settings (15).

The adaptation of Pediatric Acute Respiratory Distress guidelines to RLS provides an example of this issue (16). We reviewed the case of a 3-year-old patient with SAM diagnosed with pediatric acute respiratory distress syndrome (PARDS), who remained hypoxemic despite a ventilator positive end-expiratory pressure (PEEP) of 10 cm H_2_O and an inspired oxygen fraction (FiO2) of 80%. According to the PARDS guidelines, an increase in PEEP might be warranted from a respiratory perspective (16). However, the clinical team expressed concern about the hemodynamic effects of this in an unstable patient with limited monitoring capabilities. Moreover, there is a paucity of data on the optimal ventilation strategy for children with PARDS and SAM. This discussion reinforced the importance of teaching that emphasizes non-invasive methods for monitoring patient hemodynamics, such as clinical examination, frequent vital-sign measurements, and assessment of end-organ function. Careful attention to these parameters allows the clinical team to continue to optimize ventilation and hemodynamic strategy in the absence of invasive monitoring.

The management of patients with elevated intracranial pressure (ICP) due to trauma or infection is a frequent topic of discussion, as many of the clinical sites lack ready access to pediatric neurosurgeons, mechanical ventilation, and invasive ICP monitoring. Within these constraints, a reconsideration and codification of alternative methods for monitoring and managing these patients is essential. In the case of an infant with a large subdural empyemaand a low Glasgow Coma Scale (GCS) score, many guidelines recommend immediate endotracheal intubation and invasive intracranial pressure monitoring (17). Due to resource constraints, however, this patient was managed without invasive monitoring or mechanical ventilation and ultimately underwent a life-saving craniotomy with non-invasive ventilation and local analgesia. This patient had a favorable outcome. In the ensuing discussion, we reviewed the utility of non-invasive measurements of ICP, most notably optic nerve sheath diameter (ONSD). ONSD is measured with an ultrasound machine, which most sites have access to, and has good correlation with invasive intracranial pressure monitors (18).

Throughout these discussions, we have encountered countless examples of ingenuity, adaptability and resourcefulness, whether it is repurposing equipment (central line kits used for cricothyrotomies or a variety of catheters employed to implement peritoneal dialysis) or off-label medication use. Aside from often providing examples of decisive, lifesaving actions, these cases have highlighted the need for site-appropriates guidelines that combine established evidence-based medicine with local realities of resource constraints. In addition, several case discussions have underlined the need for procedural training of pediatric intensivists and emergency physicians beyond the typical repertoire of similar specialists in HIC. Some examples of this include performing Burr hole procedures for life-threatening epidural hemorrhage and performing emergent cricothyrotomies or tracheostomies using existing materials.

## Future directs: implications and opportunities

Case presentations in the forum described here allow for a deeper understanding of the perspectives and experiences of providers working in resource-constrained milieus. These observations can inform education and training and guide international adaptation efforts in these settings and infuse teaching with the clinical realities. In a concrete manner, the cases themselves could also be transformed into valuable teaching tools used independently or with sessions on similar themes.

Our program has important limitations. We have not yet begun to gather data on measurable educational and clinical outcomes. Assessing the impact of these sessions on knowledge retention and understanding will be an important first step. More importantly, and challenging, would be studying their effects on behavior and on patient care and outcomes. Yet this, after all, is our goal as pediatric critical care global clinician educators. Fortunately, case presentations themselves are an important window into practice and outcomes.

Finally, case presentations underscore the potential and need to develop and study locally grounded, alternative approaches to caring for sick children. We have come across numerous examples that warrant further assessment, such as the use of intravenous magnesium for pulmonary hypertension, the use of aminophylline for renal failure, and the utility of point-of-care ultrasound (POCUS) to assess intracranial pressure or to verify endotracheal tube placement. Alternative and innovative approaches offer opportunities for research, training, and collaboration that can be reinforced by this global network of clinician educators.

## Conclusion

Case-based education, an established teaching approach for adult learners, is both feasible and well-received. Case presentations, in the context of remote pediatric critical care education, allow a deeper appreciation of the circumstances in which colleagues provide care for critically ill children in RLS and, hence, more meaningful teaching. This format encourages bidirectional learning, reassessment of differing approaches, and resourcefulness. Finally, case presentations in this setting may strengthen global bonds and foster collaboration among international faculty members.

## Data Availability

The raw data supporting the conclusions of this article will be made available by the authors, without undue reservation.
